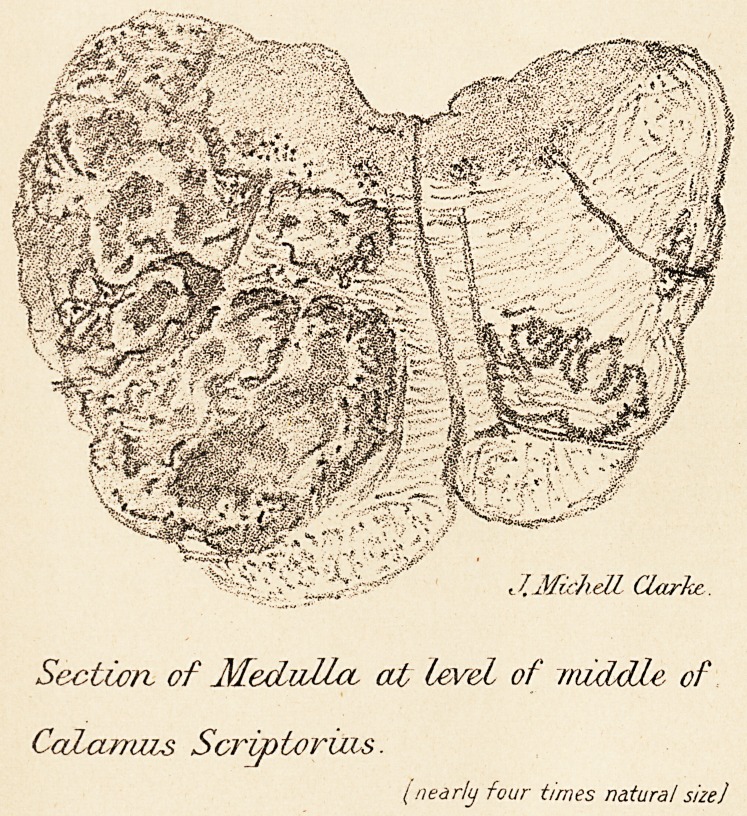# Tubercle of Medulla Oblongata

**Published:** 1889-09

**Authors:** J. Michell Clarke

**Affiliations:** Assistant-Physician to the Bristol General Hospital


### TUBERCLE OF MEDULLA OBLONGATA. By J. MICHELL CLARKE, M.B., M.A., Assistant-Physician to the Bristol General Hospital.

F. W., a boy of 8 years of age; there was some evidence of phthisis in the family history.

His mother attributed the illness to a fright twelve months ago, when the boy became afraid to go to bed by himself. After a short time he began to vomit constantly every morning, the vomit consisting of altered food. He attended for a few weeks as an out-patient, and then was admitted into the Hospital, as the vomiting and wasting continued unchanged.

January 30th. — On admission he was pale and emaciated; he vomited every morning before breakfast, but not again during the day. On examination no abnormal physical signs could be discovered in the chest or abdomen.

Vision was good; there was no affection of the special senses. There was no headache, nor giddiness, no paralysis, nor any convulsions nor fits of any kind. The pupils were equal, and acted to light and accommodation. Pulse, 80—90. Temperature never higher than 99°. Urine normal. The vomiting continued until one month after admission, when it ceased; he gained flesh and strength, and was able to take solid food.

On March 7th the left knee became full of fluid, which subsided gradually and disappeared in about three weeks.

On March 13th the vomiting returned, with troublesome hiccough, and occurred immediately after taking food at any time in the day. So long as he took no food, there was no sickness. On March 26th he brought up a large quantity of black tarry fluid. The vomiting continued with rapid emaciation until his death, which took place on April 10th. During the last three weeks the pulse was rapid, from 120—140, and for the last three days he could not bear the light; the night before death he was delirious, and did not recognise the nurse. There was no pain throughout the illness.

At the autopsy, six hours after death, the lower lobe of the left lung was adherent to the diaphragm. At about its centre near the under surface was a nodule about the size of a hazel nut, of firm consistence, and of white colour, with patches of dark pigment. It was surrounded by a zone of infiltrated and inflamed lung-tissue, in which were seen some tubercles. Apices and rest of lungs normal.

The left side of the medulla oblongata appeared swollen to twice the size of the right, and, on cutting sections through it, there was found a mass of about the size of a large hazel nut. It extended from about an $\frac{1}{8}$th of an inch above the junction of the pons and medulla to just above the commencement of the pyramidal decussation below. Its greatest diameter was about half an inch at the middle of the fourth ventricle, gradually becoming smaller towards its upper and lower extremities. At its lower end it, however, somewhat increased in size, and here there was also a small nodule on the right side in the postero-lateral region. The tumour infiltrated at the middle of the fourth ventricle nearly the whole of the anterior and lateral regions, the anterior pyramids being clearly separated from it in front, and the grey matter of the central regions and of the eminentia teres being partly infiltrated, but chiefly pushed upwards and towards the middle line. In the lower part of the pons the growth lay just above the olivary body, and in a direction inwards and upwards.

The middle and upper part of the pons, and all the other parts of the brain, were quite normal. The tumour consisted of a central caseous area, fairly firm in consistence, white in colour, with patches of dark pigment, surrounded by a zone of infiltration, and in this microscopical examination showed numerous giant-cells. The nucleus of the pneumogastric appeared to be chiefly affected by the deposit, the other nerve nuclei and fibres being pushed aside by it. The drawing shows the extent of the growth at the level of the middle of the calamus scriptorius, the commissure is pushed over to the right, and the pyramidal tract downwards. The olivary body is totally destroyed at this level; but remains of it could be detected, both above and below this.

The liver was fatty; the stomach and other organs normal.

The most remarkable feature of the case is the absence of symptoms, beyond the vomiting, to indicate the nature of the patient's complaint. It would seem improbable that a mass of such relatively large size could occupy a region so important to life without producing more widespread effects. The tumour, however, seems to have pushed aside, to have infiltrated without destroying, the nerve-centres in its neighbourhood. Thus the pneumogastric nucleus was the only one affected to such an extent as to interfere with the due performance of its function; and the effect on this was rather one of irritation than of paralysis. The hypoglossal nerve-roots and the pyramidal tracts were pushed aside. As a general rule, tuberculous deposits, from their greater tendency to early caseation, rapidly break down and destroy the tissue in which they

J. Michell Clarke.

*Section of Medulla at level of middle of Calamus Scriptorius.*

*(nearly four times natural size)*

grow; while carcinomatous and sarcomatous growths may infiltrate an organ without causing sufficient destruction of the histological elements to prevent their performing their functions.

The boy died from the long-continued vomiting and consequent emaciation, without any of the localising symptoms that one would have expected from a tumour in this situation. It is interesting to note the entire absence of abnormal phenomena connected with the vaso-motor and respiratory centres and of sugar from the urine.

The characters of the deposit in the left lung indicate that it was antecedent in date to that in the medulla: the course of events being probably the lodging of an embolus —bacillary or otherwise—which had passed up the left vertebral artery from the nodule in the lung. From the position of the tubercular mass in the centre of the lower lobe of the left lung, surrounded on all sides by healthy lung-tissue, its existence could not have been diagnosed during life. The case affords another instance of the occasional liability in pulmonary disease for secondary intracranial deposits to occur, Apart from this accident, the lesion in the lung appeared to have become inactive.

## Figures and Tables

**Figure f1:**